# Characterization of a Deswapped Triple Mutant Bovine Odorant Binding Protein

**DOI:** 10.3390/ijms12042294

**Published:** 2011-04-04

**Authors:** Eugenia Polverini, Paolo Lardi, Alberto Mazzini, Robert T. Sorbi, Conti Virna, Roberto Ramoni, Roberto Favilla

**Affiliations:** 1 Department of Physics and CNISM, University of Parma, V.le Usberti 7A, Parma, Italy; E-Mails: eugenia.polverini@fis.unipr.it (E.P.); paololardi@libero.it (P.L.); alberto.mazzini@fis.unipr.it (A.M.); roberttibor.sorbi@fis.unipr.it (R.T.S.); 2 Department of Animal Production, Veterinary Biotechnologies, Food Quality and Safety, University of Parma, V. del Taglio 8, Parma, Italy; E-Mails: virna.conti@unipr.it (C.V.); roberto.ramoni@unipr.it (R.R.); 3 Department of Biochemistry and Molecular Biology, University of Parma, V.le Usberti 23A, Parma, Italy

**Keywords:** odorant binding proteins, unfolding/refolding, molecular dynamics

## Abstract

The stability and functionality of GCC-bOBP, a monomeric triple mutant of bovine odorant binding protein, was investigated, in the presence of denaturant and in acidic pH conditions, by both protein and 1-aminoanthracene ligand fluorescence measurements, and compared to that of both bovine and porcine wild type homologues. Complete reversibility of unfolding was observed, though refolding was characterized by hysteresis. Molecular dynamics simulations, performed to detect possible structural changes of the monomeric scaffold related to the presence of the ligand, pointed out the stability of the β-barrel lipocalin scaffold.

## 1. Introduction

OBPs belong to the kernel lipocalin family (a member of the calycin superfamily) [1,2], which, despite the low degree of sequence similarity among its members, is characterized by a well conserved eight-stranded antiparallel β-barrel [3,4]. These proteins are mainly involved in the transport of hydrophobic molecules, as well as in the formation of large soluble complexes with other macromolecules through interactions with the so called “omega loop”, that contains a 3_10_ helix [5,6] and connects the βA–βB strands. Despite, or perhaps thanks to, their broad substrate specificity, OBPs probably play a fundamental role in the olfactory process [7], not only to carry odours from the air to the olfactive receptors through the aqueous layer of the nasal mucosa, but also to withdraw them, after signal transduction or in case their concentration is too high [8]. Besides, the binding capacity and chemical resistance of OBP for alken-aldehydes derived from peroxidation of fatty acids allows us to hypothesize a role of scavenger for low MW toxic compounds (150–300 Da) produced in nasal tissue in consequence of oxidative stress [5].

The study of protein structure-function relationships has been largely facilitated by the development of site directed mutagenesis, that offers the possibility to modify the sequence of any protein at will and to understand, at least in principle, the role played by the mutated residues from their effect on the structural and functional properties of that protein [9]. This strategy was applied to bovine odorant binding protein (bOBP), a swapped dimeric protein [10,11], to turn it into a functional monomer at neutral pH. To this aim, two modifications were made to bOBP: first, a Gly residue was inserted after Lys121 (Gly121+) [12] in the so called “hinge loop”, that connects the barrel to the *C*-terminal α-helix. This insertion was made to increase the hinge-chain flexibility of bOBP that is considered to be responsible of the monomeric state of porcine odorant binding protein (pOBP) [13]. Though the β-barrel topology, common to all lipocalins, represents a very good example of an evolutionary conserved stable structure [14] and therefore the mutant protein is likely to show enough stability, the presence of a disulfide bridge turns out to be necessary to protein stability, as already observed with a single mutant pOBP, where the SS bridge was removed by site-directed mutagenesis [15]. Therefore, a disulfide bridge, linking the *C*-terminal region to the barrel surface, was inserted by substituting Trp64 and His155 with two Cys residues, in the same position where they are in pOBP. A crystal structure of this mutant protein that has been abbreviated to GCC-bOBP from “Gly-Cys-Cys-bOBP”, was recently resolved [16]. More recently, phosphorescence, FTIR and short time scale MD studies mainly reported on the thermal stability of this protein [17–19].

The main goal of this work was to investigate and characterize the stability and functional properties of the OBP scaffold, by means of both computational and spectroscopic techniques, against guanidinium chloride (GdnHCl) concentration or pH conditions, in the presence and absence of AMA as ligand. The properties of this monomeric mutant of bOBP were compared to those of the wild type bovine and porcine homologues, in view of possible utilizations of OBPs in biotechnological applications, e.g., as a scaffold for the production of protein affinity reagents for small hydrophobic molecules [16] and/or as sensitive elements in biosensor systems for a number of compounds (narcotics, explosives, toxic agents, *etc*.) [20].

## 2. Results and Discussion

### 2.1. Stability and Functionality of GCC-bOBP at Neutral pH

**Figure 1.**
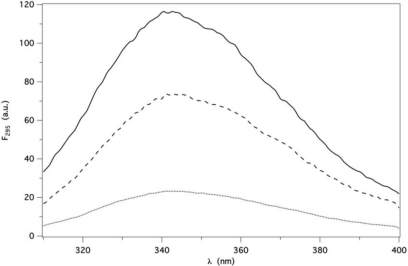
Fluorescence spectra of bOBP (—), GCC-bOBP (---) and pOBP (....). Each protein was 1 μM (subunit concentration) in P buffer at pH 7.

GCC-bOBP has two Trp residues (W17 and W133) per subunit, whereas wild type bOBP and pOBP have three (W17, W64 and W133) and one (W17 or W16 in the porcine sequence numbering), respectively. The fluorescence spectra of the three proteins, collected under identical conditions differ in intensity, but have a similar shape (λ_max_ = 346 ±1 nm and FWHH = 55 ±2 nm, Figure 1).

Assuming that the homologous Trp residues have the same fluorescence quantum yield, independently of the protein they belong to, as suggested by their very similar environments in the crystals, the relative contribution of each Trp residue to the total fluorescence can be estimated by simply comparing the total fluorescence intensity of each protein, collected under identical conditions (Table 1).

It turns out that Trp133 and Trp64 of bOBP, though more exposed to the solvent, are more fluorescent than Trp17, hidden inside the β-barrel, by about a factor two. This can be explained considering that Trp17 is probably largely quenched by the nearby residue Lys121 [21] and as fluorescent as free Trp under the same conditions (data not shown).

**Figure 2.**
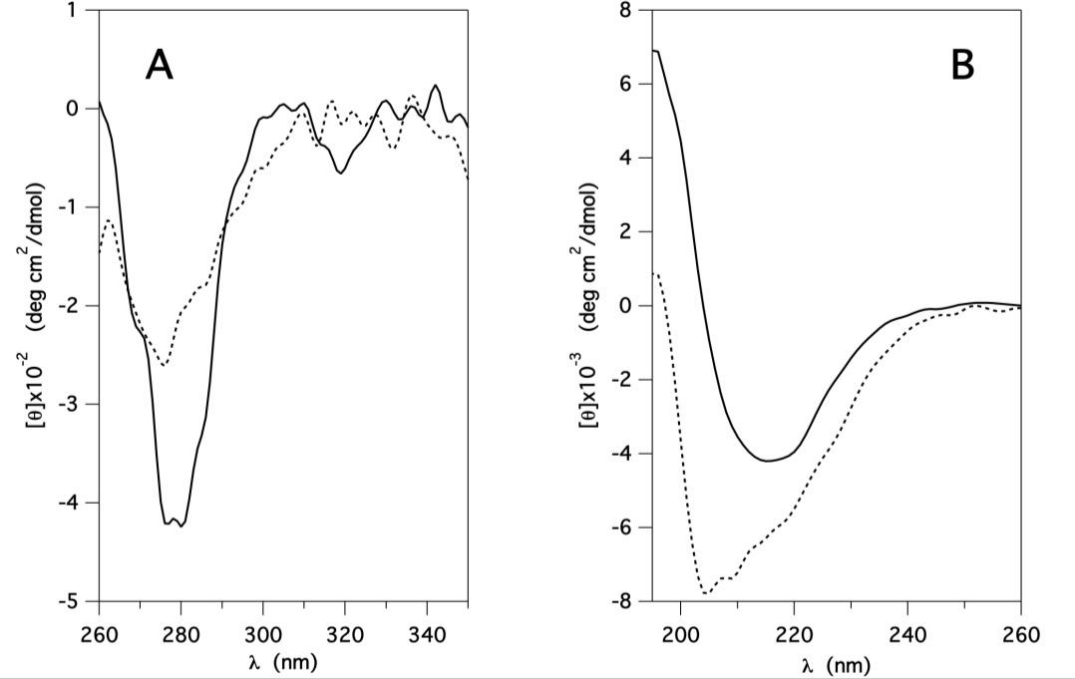
(**A**) Near UV and (**B**) far UV molar ellipticity of GCC-bOBP in P buffer at pH 7 (continuous line) and pH 1 (dotted line). Protein concentration: 5 μM in (B) and 10 μM in (A).

The near and far UV CD spectra of GCC-bOBP, with a negative trough at about 280 nm, a negative trough at 215 nm and a peak below 200 nm (continuous curves in Figure 2a,b) are also very similar to those of the two wt OBPs [22,23], strongly confirming, as expected, the maintenance of the β-barrel crystalline structure in solution.

### 2.2. Binding of AMA to Native GCC-bOBP

Considering that OBPs bind one AMA molecule inside the β-barrel stoichiometrically and with a good affinity (*K_d_* ≈ 1 μM), this fluorescent ligand is often used to monitor their functional state [24]. A binding study with variable AMA and constant GCC-bOBP concentrations was thus performed, to see how much the functional properties of GCC-bOBP and wt OBPs are related.

**Figure 3.**
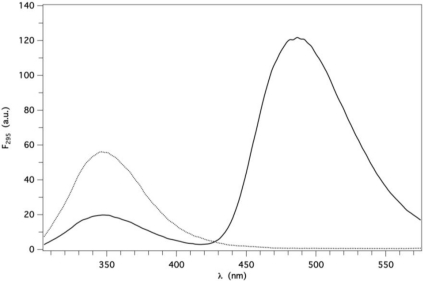
Fluorescence spectra of 5 μM GCC-bOBP alone (dotted line) and in the presence of 50 μM AMA (continuous line) in P buffer at pH 7. Excitation at 295 nm in both cases.

A very large increase of AMA fluorescence, together with a very large spectral blue shift, was indeed observed in the presence of GCC-bOBP, upon ligand excitation at 350 nm. By plotting the fluorescence intensity at 487 nm as a function of AMA concentration, at constant protein, a hyperbolic binding curve was derived (data not shown), from which *K_d_* = 5.0 ± 0.2 μM was obtained using Equation (A.3), a slightly higher value than that previously found by us (≈1 μM) for the two homologous wt proteins [22,23]. Since the fluorescence spectrum of GCC-bOBP is largely overlapped to the absorbance spectrum of AMA, FRET effects were expected upon binding, as already observed with pOBP [23]. A large protein fluorescence quenching and a concomitantly large AMA fluorescence increase were indeed observed with 5 μM GCC-bOBP in the presence of 50 μM (saturating) AMA, compared to the absence of ligand, by exciting at 295 nm (Figure 3).

**Figure 4.**
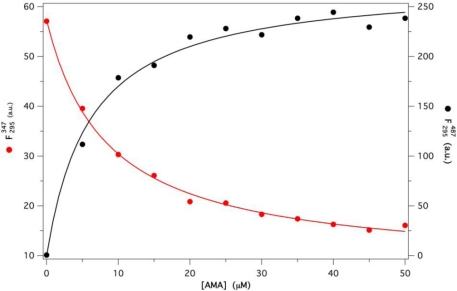
$F_{295}^{347}$ : FRET induced quenching of GCC-bOBP fluorescence at 347, with excitation at 295 nm, as a function of AMA (red dots and line); $F_{295}^{487}$ : FRET induced enhancement of AMA fluorescence at 487, with excitation at 295 nm, as a function of AMA (black dots and line). [GCC-bOBP] was fixed at 5 μM, while [AMA] varied from 0 to 50 μM in P buffer pH 7. Protein and ligand data were fitted according to Equations (A.1) and (A.3), respectively.

From the amount of protein fluorescence quenching, a FRET efficiency of ∼0.6 was calculated using Equation (1a). The large protein fluorescence quenching observed can be attributed to FRET only, since no appreciable inner filter effects occur (absorbance at 295 nm <0.1 at 50 μM AMA). Binding of AMA could thus be investigated not only by the increase of the AMA fluorescence upon excitation at 350 nm, but also by the simultaneous detection of FRET-dependent fluorescence changes of both GCC-bOBP and AMA, upon excitation at 295 nm ( $F_{295}^{347}$ and $F_{295}^{487}$, respectively). A substantially identical value of *K_d_* (4.8 ± 0.2 μM) was obtained from both series of data, shown in Figures 4, once fitted to Equations (A.1) and (A.3), respectively. This result is also in excellent agreement with that derived from direct AMA fluorescence excitation at 350 nm.

As far as FRET efficiency is concerned, a Förster distance *R*_0_ ≈ 50 Å would be derived from Equation (3), assuming complete rotational freedom around the two chromophores (*k*^2^ = 2/3) and *Φ_D_* = 0.1 for GCC-bOBP, as obtained with the comparative method [25] using *Φ* = 0.14 for Trp in water [26]. With *R*_0_ ≈ 50 Å, a value of *R* ≈ 45 Å would result from Equation (2), representing the average distance between the two Trp residues of GCC-bOBP and AMA, under the assumption that both Trp residues contribute equally to FRET. However, looking at the tertiary crystal structure of the mutant protein, assuming that AMA keeps the same position it has in the bOBP-AMA crystal, the resulting *R* value is much shorter (12 Å for AMA-W17 and 18 Å for AMA-W133). A plausible explanation for this discrepancy will be given below under the FRET paragraph.

### 2.3. GdnHCl-Induced Unfolding and Refolding of GCC-bOBP

**Figure 5.**
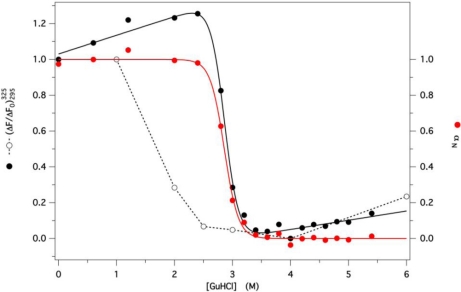
Dependence of the protein fluorescence on denaturant concentration. ${\left(\Delta F/\Delta F_{0}\right)}_{295}^{325}$ is the ratio between the fluorescence intensity measured at each denaturant concentration examined and the initial value at 325 nm, with excitation at 295 nm, (unfolding: black dots; refolding: empty dots). Red dots refer to the dependence of the molar fraction of the native protein *α_N_* on denaturant concentration. All data shown here were taken 2 h after the beginning of each of the two processes. Black and red lines are the best fit curves obtained using equations reported under the experimental section.

The protein fluorescence intensity at 325 nm, with excitation at 295 nm, was monitored at several GdnHCl concentrations (between 0 and 6 M). The ratio between actual and initial values ${\left(\Delta F/\Delta F_{0}\right)}_{295}^{325}$ was plotted for both unfolding and refolding experimental data as a function of denaturant concentration, to check for reversibility. Hysteresis between unfolding and refolding data was observed at short times (e.g., after 2 h from dilution of the denatured protein, as shown in Figure 5), with complete refolding observed only at low denaturant concentration. At longer times, refolding data shifted progressively to the right, in contrast to the unfolding data, that remained stable, but complete overlap occurred only at much longer times (data not shown). This behavior demonstrates the reversibility of the folding process, thus allowing us to derive the thermodynamic folding parameters (*m* and *C*_1/2_) using Equation (4). A similar pattern was also obtained in the presence of 50 μM AMA, monitoring the ligand fluorescence (data not shown). The pattern of *α_N_*, the residual degree of native protein, as a function of the denaturant concentration *C*, was then calculated fitting the unfolding data to Equation (5), to account for the linear dependence of the native and denatured protein fluorescence upon denaturant concentration (Figure 5, black dots and line).

The best fit unfolding parameters are reported in Table 2, together with those previously obtained for the two bovine and porcine wt OBPs [23,27] for the sake of comparison. The larger value of the standard free energy of unfolding for GCC-bOBP in buffer (Δ*G*°*_U_*) is apparently due to both higher cooperativity and *C*_1/2_ values, compared to the values of these parameters obtained for the wt proteins.

**Figure 6.**
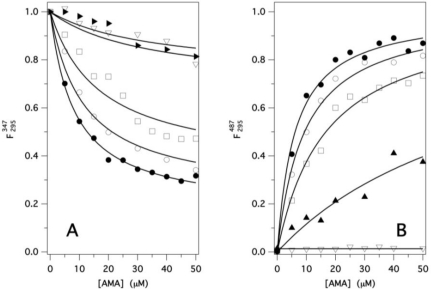
(**A**) Quenching of $F_{295}^{347}$, the fluorescence intensity of GCC-bOBP (emission at 347 nm with excitation at 295 nm); and (**B**) Enhancement of $F_{295}^{487}$, the fluorescence intensity of AMA (emission at 487 nm with excitation at 295 nm), as a function of AMA concentration (0–50 μM) at several GdnHCl concentrations (•: 0, ○: 1, □: 2.5, ▴: 3, ∇: 4 M). [GCC-bOBP] = 5 μM, P buffer pH 7. Protein fluorescence values are normalized to those in the absence of AMA. Protein and ligand data were fitted according to Equations (A.1) and (A.4) in the Appendix, respectively, to obtain *K_d_* and *F*_∞_ values as a function of denaturant concentration.

Unfolding and refolding were also investigated by following the changes of AMA binding capability at several denaturant concentrations (between 0 and 4 M), by simultaneously recording the protein and ligand FRET-dependent changes ( $F_{295}^{347}$ and $F_{295}^{487}$), respectively (Figure 6A,B**)**. The best fit *K_d_* values were derived with good accuracy from each protein and ligand binding curve using Equations (A.2) and (A.4), respectively, and are invariant (*K_d_* ≈ 5 ±1 μM) in the whole pre-unfolding region (0–2.5 M GdnHCl). This result points out that the protein functionality remains practically unaffected by the presence of denaturant as far as the β-barrel structure is preserved. Above this concentration, *K_d_* values cannot be derived with accuracy because of progressive rapid protein denaturation. Actually, the increase of AMA fluorescence, due to specific binding into the protein β-barrel, is rapidly abolished (Figure 6B, lowest curve), while the protein fluorescence keeps decreasing with AMA concentration, though with a reduced amplitude, above 3.5 M GdnHCl (Figure 6A, uppermost curve). In fact, the red shifted fluorescence spectrum of the completely unfolded GCC-bOBP in the presence of AMA resulted about 20% less intense compared to that in the absence of the ligand, whereas the fluorescence spectrum of AMA, when excited at 295 nm, was practically unaffected by the presence of the unfolded protein. The residual protein fluorescence quenching cannot be due to FRET, since this effect occurs only in the presence of the specific binding of AMA inside the native protein cavity. A reasonable explanation of this effect can be found in an unspecific interaction of AMA with solvent exposed Trp residues, present on the surface of the denatured protein.

### 2.4. Protein Fluorescence Lifetime

The fluorescence decay measurements can give more detailed information on protein Trp microenvironment compared to steady state fluorescence spectra, for example to decide whether FRET is present or not in a given system, as described by Equation (1b). Fluorescence decay curves of GCC-bOBP (10 μM) were collected in the absence and in the presence of AMA (100 μM) under native (P buffer, pH 7) conditions, upon excitation at 289 nm and emission at 350 nm (bandwidth 10 nm). As a control, a decay curve of 100 μM AMA was also collected in the same conditions and found very similar to that of P buffer alone. As reported in Table 3, the deconvoluted decay curves were best fitted by two exponentials in the absence of AMA and by one exponential in the presence of AMA. Interestingly, the lifetime observed in the presence of AMA is very similar to the shortest lifetime observed in its absence, suggesting that one of the two Trp residues may be totally quenched by the ligand.

### 2.5. Molecular Dynamics Simulations at Neutral pH

At first, a 20 ns simulation on the X-ray resolved crystal structure was performed in the absence of ligand, to test the structure stability.

**Figure 7.**
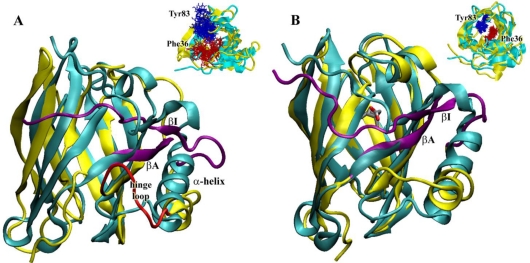
(**A**) Superimposition of the crystal (cyan) and the last structure collected after 20 ns of MD simulation at neutral pH (yellow, with the βA and βI strands in purple and the hinge loop in red); (**B**) Superimposition of the crystal (cyan) and the last structure collected after 20 ns of MD simulation in the presence of ligand at neutral pH (yellow, with the βA and βI strands in purple). Insets: all the positions in the trajectory (collected every 40 ps) of the two “door” residues, Tyr83 (blue) and Phe36 (red), highlighted in a stick representation.

From a visual inspection of the MD trajectory, the first evidence is the deformation of the barrel at the end closed by the Ω-loop, in particular at the Ω-loop itself, at the βC–βD loop and at the βA and βI strands that seem to be dragged by the *C*-terminal α-helix (Figure 7A). In fact, also the hinge loop connecting the α-helix to the barrel is highly deformed, with a partial unfolding of the helix *N*-terminal. However, the barrel supersecondary structure, with its network of hydrogen bonds, remains stable on the whole, in particular in the core region, delimited by the evolutionarily conserved regions (*i.e*., the first part of βA, the turn between βF and βG and the *C*-terminal end of βH [14]).

As already observed in our previous results regarding MD simulations on the monomeric (acidic and neutral) structure of bOBP [27], the movements of the Ω-loop and of the βE–βF loop, that contains the two “door” residues Tyr83 and Phe36 and hypothesized to regulate the access to the binding site, allow the opening of the barrel entrance [23,27,28]. In particular, they move back and forth opening and closing repeatedly the barrel access, ready to receive the ligand as highlighted by the spreading of the positions of the two residues during the whole trajectory (Figure 7A, inset). To check if the observed changes could be influenced by the presence of the ligand into the structure and taking into account that the experimental data were obtained in the presence of the ligand 3, 6-bis(methylen)decanoic acid, found after the purification procedure (see under Materials and Methods and [16]), another 20 ns MD simulation was subsequently run on the same structure in the presence of the co-crystallized ligand inside the barrel.

This simulation therefore could be useful to see if the ligand has a role in changing the flexibility of some structural regions, identifying the key regions involved, directly or indirectly, in the ligand binding or release.

The results point out a reduced flexibility of the whole structure, which remains very stable around the crystal positions (Figure 7B). In particular, the presence of the ligand keeps the Ω-loop closed and the residues that were hypothesized to regulate the access to the binding site (Phe36 and Tyr83) [29] remain about the same positions during the whole trajectory (Figure 7B, inset). These results are in agreement with those already observed [18], and our much longer simulation time scale better underlines the straightforward behavior in the presence of ligand, in particular of the regions involved in the β-barrel access regulation and ligand uptake (and E–F loops, with Phe36 and Tyr83 doors), as also observed with the two wt proteins [30]. This behavior can be interpreted as a slowdown of the dynamics of the “doors” induced by the bound ligand, as a means to prolong the residence time inside the barrel, as already proposed for the intestinal fatty acid-binding protein [31] and the retinol-binding protein [32].

Nevertheless it is evident, also in this case, that a slight deformation of the barrel in the region of the A and I strands, increases the distance between A and B causing a slight enlargement of the binding cavity. This structural feature could explain the experimentally observed increase of *K_d_* for AMA with respect to that for the wild type proteins, pointing out how minor structural rearrangements can affect protein functionality. This result also suggests the important role of the S-S bridge to link the *C*-terminal region to the barrel structure.

### 2.6. Fret Efficiency Determination

The change of the fluorescence lifetime of native GCC-bOBP in the presence of AMA confirms the occurrence of FRET. In fact, only one lifetime of 2.8 ns was observed in the presence of AMA, compared to two lifetimes (2.9 ns and 8 ns) in its absence. The FRET efficiency, derived from Equation (1b) using a weighted average lifetime of 6.85 ns [33], was 0.6, in quantitative agreement with the value derived from the fluorescence spectra of the protein in the absence and presence of AMA. A very similar result was also observed with wild type pOBP, where just one Trp residue (Trp16 in porcine sequence numbering) is present [23].

The efficiency observed for the pOBP-AMA complex (60%) is too little for a residue completely free to rotate, considering the very short distance from AMA bound inside the barrel. This result was, in fact, explained as due to a hindered rotation of Trp17, as suggested by inspection of the protein structure. In the case of the GCC-bOBP-AMA complex, a very similar FRET efficiency was also observed, though there are two Trp residues in this protein. Assuming a similar quantum yield of Trp17 in the two proteins, it follows that Trp133 should also be 60% quenched. However, though Trp133 is more distant from AMA than Trp17 (≈18 and ≈12 Å, respectively), the actual distance R is still much lower than R_0_, therefore the experimental value of E can again be explained only assuming that Trp133 is also rotationally restricted, as Trp17. This conclusion is indeed supported by the MD time pattern that highlights only minor oscillations of this residue about the plane of the ring (not shown).

### 2.7. GCC-bOBP Stability at Acidic pH

**Figure 8.**
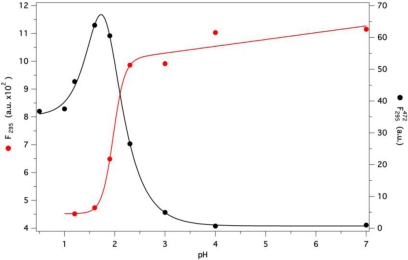
Total fluorescence intensity of the protein alone (red dots and line) and ANS fluorescence intensity at 487 nm in the presence of the protein (black dots and line) as a function of pH (excitation at 295 nm in both cases). The protein was 5 μM and ANS 20 μM in P buffer.

The stability of GCC-bOBP under acidic conditions was also investigated. A large decrease of fluorescence intensity, with a small blue shift (5 nm), was observed in the protein spectrum at pH 1, with respect to that at neutral pH (data not shown). The pH-dependence of the protein fluorescence shows a sharp transition below pH 3, with midpoint at pH ≈ 2.0 (Figure 8, red dots and line), pointing out a considerable pH stability of GCC-bOBP. Only at pH 1, the protein undergoes complete acid denaturation and loss of AMA binding capacity (data not shown).

Protein CD spectra in the near and far UV regions were also collected at pH 1 (dotted line in Figure 2A). The near UV CD spectrum shows a trough near 280 nm with a considerable loss of intensity, suggesting an increased flexibility of the aromatic residues with respect to that at pH 7. The far UV CD spectra (dotted line in Figure 2B) also differ considerably at the two pHs: whereas at pH 7, the peak below 200 nm and the trough at 215 nm are consistent with the presence of a large β-structure content, the large shift towards shorter wavelengths, observed at pH 1, is due to the protein acid denaturation.

In order to get more insight into the protein acidic structure, ANS, a dye frequently used to probe the presence of molten globule-like states [34], was added to the protein. At pH 2 the protein fluorescence results largely quenched (about 60%) compared to that at pH 7, whereas ANS fluorescence shows a large enhancement and a large blue shift (from 520 to 470 nm), suggesting the presence of FRET, also observed with AMA under native conditions. The pH dependence of the ANS fluorescence intensity at 472 nm (Figure 8, black dots and line) shows a sharp transition below pH 3, with a maximum intensity slightly below pH 2. This result suggests the formation of a molten globule-like state in the pH range between 2.5 and 1.5, before complete acid denaturation takes over. Interestingly, it has recently been pointed out that ANS fluorescence intensity peaks may also derive from aggregation of partially folded states, in the presence of low GdnHCl concentrations [35]. Though this possibility was not investigated, it seems rather unlikely because no GdnHCl was present and the protein is highly positively charged at low pH (pI 4.9, calculated from its aminoacid composition).

### 2.8. Molecular Dynamics Simulations at Acidic pH

**Figure 9.**
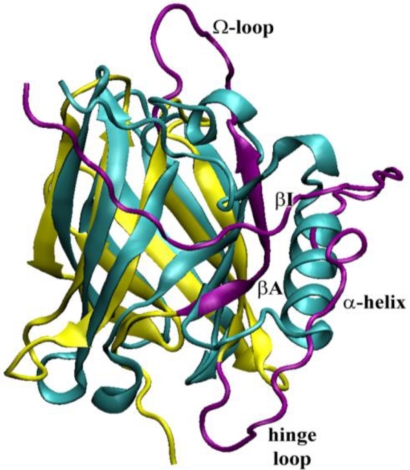
Superimposition of crystal structure (cyan) and structure collected after 30 ns of MD simulation at acidic pH (yellow, with *C*-terminal-helix, -loop and A and I strands in purple).

Starting from the crystal structure, a third MD run was performed at very low pH (<pH 3) and in the absence of ligand, assuming that it does not bind to the protein under these conditions, as indicated by the experimental results. Even if after 30 ns no stable structure was yet reached, a trend towards a partial loss of secondary structure, involving the *C*-terminal helix, the loop and the I strand, is observed, in agreement with the experimental results. In addition, the barrel starts deforming, particularly in the region of the strands A, E, F, G and H, with two big enlargements between strand A and B and between strand D and E (Figure 9). The binding site, that in the crystal is a closed cavity containing the ligand, becomes an open pocket exposing hydrophobic residues, in agreement with the experimental results obtained with ANS. After 30 ns simulation, the barrel H-bonds network is about 80% preserved, thus preventing the complete exposition of Trp17, which anyway never occurs at acidic pH, as already discussed above.

## 3. Experimental Section

### 3.1. GCC-bOBP

The triple mutant GCC-bOBP was prepared according to the procedure recently described [16]. The protein purity was checked by SDS-PAGE electrophoresis. Protein concentration was estimated by absorbance, assuming *ɛ*_280_ = 18,300 M^−1^ cm^−1^, as derived from the aromatic residue content. Wt bOBP and pOBP proteins were prepared according to the original procedures [13,36].

### 3.2. Spectroscopic Measurements

Fluorescence measurements were made on an LS-50 spectrofluorometer (Perkin Elmer), with excitation at 295 nm and 350 nm for protein and AMA fluorescence, respectively, and 5 nm excitation and emission bandwidth, was used throughout at 20 °C. The emission spectra were run at 60 nm/min with point acquisition every 0.5 nm, using a precision microcuvette with 3 mm excitation and emission pathlength (Hellma 105.251). Fluorescence spectra were corrected for baseline and inner filter effects, where necessary [25].

Circular dichroism measurements were made on a J-715 Jasco spectropolarimeter, using either 10 mm (near UV) or 2 mm (far UV) cell pathlengths.

### 3.3. Fluorescence Resonance Energy Transfer

Binding of AMA to GCC-bOBP was found to be characterized by fluorescence resonance energy transfer (FRET). This gave us the opportunity to correlate simultaneously the functional and structural properties of this protein as a function of GdnHCl. FRET efficiency *E* can be calculated by either equation:

(1a) $$E=1-\frac{F_{DA}}{F_{D}}$$

where *F_D_* and *F_DA_* represent the donor *D* fluorescence intensity in the absence and in the presence of saturating acceptor *A*, respectively, or:

(1b) $$E=1-\frac{\tau_{DA}}{\tau_{D}}$$

where *_DA_* and *_D_* represent the average fluorescence lifetime of the donor in the presence and absence of the acceptor, respectively [33].

The average *D-A* distance, *R*, can be derived from:

(2) $$R=R_{0}{\left[\frac{E}{1-E}\right]}^{\overset{1}{\;}/\underset{6}{\;}}$$

where *R*_0_ is the Förster radius, *i.e.*, the *D-A* distance at which *E* = 0.5. In turn, *R*_0_ is related to the spectral properties of the two chromophores by:

(3) $$R_{0}^{6}=\frac{9000\cdot \text{ln}\;10\cdot k^{2}\phi_{D}}{128\pi^{5}\;N\cdot n^{4}}\;\int \;F_{D}(\lambda ){ɛ}_{A}(\lambda )\lambda^{4}\;d\lambda$$

where *k* is the orientation factor, related to the relative orientation of the two transition dipoles, *Φ_D_* the quantum yield of the donor, *N* the Avogadro number, *n* the refractive index. The integral accounts for the overlap between absorbance spectrum of the acceptor (*ɛ_A_*) and the normalized fluorescence spectrum of the donor (*F_D_*).

*R*_0_ can thus be calculated, provided the overlap integral between the fluorescence spectrum of the donor and the absorbance spectrum of the acceptor, as well as *k*^2^, is known. While the spectra of the two chromophores and *Φ_D_* are easily available, the same is not true for *k*^2^ and for this reason it is usually put equal to 2/3, corresponding to complete rotational freedom of the chromophores. However, if one of the two chromophores is a ligand strongly bound to a protein, as in our case, where the ligand AMA is deeply bound inside the barrel, this value for *k*^2^ cannot be assumed. If *R* is known, e.g., from X-ray data, the experimental value of *E*, derived from either Equation (1a) or (1b), can be used to estimate *R*_0_ from Equation (2) and, in turn, *k*^2^ from Equation (3). This is the pattern followed by us with GCC-bOBP as donor and AMA as acceptor, as described under Results and Discussion.

### 3.4. Fluorescence Lifetimes

Fluorescence decay measurements (λ_ex_ = 289 ± 10 nm, λ_em_ = 350 ±10 nm) of the Trp residues of GCC-bOBP were made either in the absence or in the presence of AMA and/or denaturant. The lifetime instrumentation used is a device assembled in our laboratory which has been described in detail elsewhere [37]. The experimental fluorescence decays were deconvoluted *versus* the instrumental response function, obtained from the light scattering of a glycogen solution excited at the same wavelength.

### 3.5. Functional Assays

Protein functionality was assayed using AMA as a reference ligand [24]. The values of *K_d_*, the dissociation constant of AMA from the complex with GCC-bOBP, were obtained from a series of fluorescence titrations at constant protein concentration *P*_0_ (5 μM) and variable ligand concentration *L*_0_ (0–50 μM) in 0.1 M phosphate buffer at pH 7.0 at different denaturant concentrations and by using Equations (A.1–A.4) reported in the Appendix. Binding assays were also performed at pH 1.5 using a 10 fold excess of AMA (50 μM) over the protein.

### 3.6. Unfolding and Refolding Measurements

Unfolding and refolding of GCC-bOBP were investigated by recording the protein emission fluorescence intensity at different times. Unfolding was achieved after a ten dilution of a 50 μM native protein solution in 0.1 M neutral phosphate buffer (P buffer) containing appropriate amounts of 6 M GdnHCl. A similar procedure was adopted to follow refolding, with the protein previously denatured in 6 M GdnHCl containing P buffer. At any given denaturant concentration, the folding parameters *C*_1/2_ and *m*, defined below, were estimated by fitting the experimental unfolding fluorescence data, since only these data represent true equilibrium values, as explained under paragraph 2.3, using Equation (4), valid for a reversible two state process, in which only the native N and denatured U species are present at equilibrium:

(4) $$F=\frac{\left(F_{N}^{0}+k_{N}C\right)+\left(F_{U}^{0}+k_{U}C\right)\text{exp}\left[\frac{-m\left(C_{\overset{1}{\;}/\underset{2}{\;}}-C\right)}{RT}\right]}{1+\text{exp}\left[\frac{-m\left(C_{\overset{1}{\;}/\underset{2}{\;}}-C\right)}{RT}\right]}$$

where *F* is the experimental fluorescence intensity, recorded at a given wavelength in the presence of denaturant at concentration *C*; since the fluorescence intensity of the native (*F_N_*), as well as the fully denatured (*F_U_*), protein is assumed to show a linear dependence on *C*, we can write: *F_N_*(*C*) = *F_N_*^0^ + *k_N_C* and *F_U_*(*C*) = *F_U_*^0^ + *k_U_C* where *F_N_*^0^ and *F_U_*^0^ are the fluorescence intensities, in the absence of denaturant (*C* = 0), of the native and fully unfolded protein, respectively; *C*_1/2_ is the denaturant concentration at half transition; *m* is the slope of the unfolding curve at half transition, a measure of cooperativity of the unfolding process.

If Δ*G_U_* is assumed to vary with the denaturant concentration according to the linear extrapolation model [9], *i.e*., Δ*G_U_(C)* = Δ*G_U_*^0^ − *mC*_1/2_, it follows that Δ*G_U_*^0^ = *m C*_1/2_ = −RT ln*K_U_*(0), where *K_U_*(0) is the unfolding equilibrium constant in the absence of denaturant.

For a simple two-state process, *α_N_*, the molar fraction of native protein, varies with *C* according to *α_N_*(*C*) = (*F* − *F_U_*)/(*F_N_* − *F_U_*). By replacing *F* with Equation (4) and using the best fit values of *F_N_*^0^, *k_N_*, *F_U_*^0^ *and k_U_*, *α_N_*(*C*) can be fitted by:

(5) $$\alpha_{N}(C)=1/\{1+\text{exp}[-m(C_{1/2}-C)/RT]\}$$

to derive alternative values of *m* and *C*_1/2_. It follows that the theoretical dependence of the unfolding equilibrium constant on GdnHCl is given by:

(6) $$KU=[U]/[N]=(1-\alpha_{N})/\alpha_{N}=\text{exp}\;[-m(C_{1/2}-C)/RT]$$

where [*U*] and [*N*] are the unfolded and native protein concentrations, respectively.

### 3.7. Molecular Dynamics

Three MD simulations were performed on the crystal structure of GCC-bOBP, obtained from the Brookhaven Protein Data Bank (pdb id code 2hlv [38]), with the GROMACS program [39] and the Gromos96 force field [40]: at neutral pH, in the absence and in the presence of the co-crystallized 3, 6-bis(methylen)decanoic acid ligand [16] and at acidic pH.

For each simulation, the protein was solvated with a pre-equilibrated water box, keeping a water layer of 8 Å around the solute molecule (corresponding to about 6500 water molecules for each system), sodium ions were added to keep the system neutral and the periodic boundary conditions were applied to the system. The two Cys residues were kept in the oxy state to form a disulfide bridge and, according to the experimental pH value, all histidine residues were kept in the neutral form. As previously stated [15], the residue Glu117 was substituted with Gly117.

An energy minimization was first performed on the whole system up to a gradient of 500 kJ/(mol nm). Afterwards, a position restrained dynamics was run for 50 ps, to let the solvent relax around the protein. Finally, a full molecular dynamics was run for 20 ns (simulations at neutral pH) or 30 ns (simulation at acidic pH) at 300 K and 1 atm, with a time step of 1 fs.

The ligand parameters were obtained by means of the PRODRG server [41]. To reproduce highly acidic conditions, all Asp and Glu residues and the *C*-terminus carboxyl group were protonated.

Structural analysis was performed with the VMD software package [42] (particularly regarding the MD trajectories) and the Swiss-Pdb Viewer program [43], whereas the H-bonds calculations were made by the DSSP program [44].

## 4. Conclusions

The unfolding experiments and MD simulations on the 3D structure of GCC-bOBP, confirmed that a stable monomeric β-barrel scaffold can be obtained by site-specific mutagenesis.

The mutant protein behaves similarly to the wt porcine and bovine homologues, as far as the structural and functional properties are concerned, with the overall maintenance of the β-barrel structure in a large range of different conditions (denaturant and pH). This supersecondary structure, with its network of H-bonds, is likely to play a structural and functional role. Besides the binding site, it contains a structural hydrophobic core that probably acts as a protein folding core since it remains stable even after a large truncation of 13 residues at the *N*-terminus, including the conserved 3_10_ helix [30]. The protein stability, enhanced by the presence of a ligand inside the barrel, suggests a role of the ligand in the regulation of the dynamics of some residues involved in the control of the accessibility to the binding cavity, particularly Phe36 and Tyr83.

The lower affinity of GCC-bOBP for AMA, compared to the wt proteins, confirms the structural rearrangement at the access to the cavity. Nevertheless, a higher stability of the mutant against chemical denaturation, compared to that of the wt bOBP and pOBP, is derived from the Δ*G_U_*° values.

The high stability of the monomeric scaffold is also confirmed by pH studies that suggest the formation of a molten globule-like state around pH 2, before complete acid denaturation.

In conclusion, we have shown that the triple mutant bovine OBP, investigated here, is slightly more stable than the wild type homologues. The observed slightly lower binding affinity towards AMA, probably due to a larger flexibility of the cavity, could be useful to investigate other ligands with higher affinity; therefore well suited for biotechnological applications, for which both these properties are highly appropriate. These data confirm that the monomeric structural frame of lipocalins with the interdomain disulfide bridge is the option that gives them greater stability. The evolutionary pathway that led to the dimeric form with domain-swapping bOBP might be due either to random mutations, which preserved the lipocalin frame within the dimer, or driven by yet unknown functional requirements, such as interaction with receptors and/or availability of novel binding sites that involve the surface of the protein at the interface between the two monomeric units.

## Figures and Tables

**Table 1.** Relative fluorescence efficiency of Trp residues of GCC-bOBP and bOBP, with respect to that of pOBP.

Protein*	**W17**	**W133**	**W64**
pOBP	1	-	-
GCC-bOBP	1	2.15	-
bOBP	1	2.15	1.88

* The fluorescence spectra were collected under identical conditions: 1 μM protein subunit concentration, P buffer at pH 7.0, excitation at 295 nm.

**Table 2.** Unfolding parameters of GCC-bOBP, bOBP and pOBP in GdnHCl.

**Parameter**	**GCC-bOBP^a^**	**bOBP^b^**	**pOBP^c^**
*C*_1/2_ (M)	2.90 ± 0.01	2.65 ± 0.03	2.37 ± 0.02
*m* (kJ mol^−1^ M^−1^)	14.3 ± 1.0	8.4 ±0.8	8.4 ± 0.4
ΔG°_un_ (kJ M^−1^)	41.5 ± 3.0	22.2 ± 2.4	19.8 ± 1.1

a from Figure 5;

b from [22];

c from [23].

**Table 3.** Fluorescence lifetime of native GCC-bOBP.

**Conditions**	**τ_1_(ns)**	**τ_2_(ns)**
N	2.9 ± 0.2 0.45 ± 0.05	8.0 ± 0.3 0.55 ± 0.05
N + AMA	2.8 ± 0.1	-

N: native protein (in P buffer, pH 7): 10 μM; AMA: 100 μM.

## Abbreviations:

AMA: 1-amino-anthracene;
ANS: 1-anilino-naphtalene sulfonate;
bOBP: bovine OBP;
CD: circular dichroism;
FRET: fluorescence resonance energy transfer;
FWHH: full width at half height;
GCC-bOBP: triple mutant (Gly-Cys-Cys) bOBP;
GdnHCl: guanidinium chloride;
MD: molecular dynamics;
NATA: *N*-acetyl-tryptophanamide;
OBP: odorant binding protein;
pOBP: porcine OBP;
P buffer: 0.1 M sodium phosphate buffer;
RMSD: root mean square deviation;
wt: wild type.

## Appendix

### Estimation of K_d_ for AMA-GCC-bOBP Complex

The fluorescence titrations (Figures 4, 6a,b) were analyzed assuming a simple 1:1 binding stoichiometry between GCC-bOBP and AMA, as observed for the wild type protein, and hence a direct proportionality between the fraction of saturation *v* and the relative change of either protein or ligand fluorescence intensity.

Upon excitation at 295 nm, the FRET dependent protein fluorescence intensity at 347 nm was recorded simultaneously to that of the ligand at 487 nm during all titrations performed at constant protein and variable AMA concentrations. For the sake of clarity, protein and ligand data are treated separately, because different fitting equations were used in the two cases.

### Protein Fluorescence

The degree of saturation *v* is related to the fluorescence change by:

$$\nu =\frac{PL}{P_{0}}=\frac{F_{0}-F}{F_{0}-F_{\infty }}$$

*P*_0_ = total protein concentration;
*PL* = protein-ligand complex concentration;
*F*, *F*_0_, *F*_∞_ = fluorescence intensities in the absence of AMA, in the presence of AMA, at infinite AMA concentration, respectively.

The saturation degree is also related to the dissociation constant *K_d_* by:

$$K_{d}=\frac{P\cdot L}{P\;L}=\frac{\left(P_{0}-P\;L)(L_{0}-P\;L\right)}{P\;L}=\frac{\left(1-\nu )(L_{0}-P_{0}\nu \right)}{\nu }$$

hence:

(A.1) $$F=F_{0}-(F_{0}-F_{\infty })\frac{(K_{d}+P_{0}+L_{0})-\sqrt{{\left(K_{d}+P_{0}+L_{0}\right)}^{2}-4P_{0}L_{0}}}{2P_{0}}$$

Since *P*_0_ is a known constant, *F*_0_, *F_∞_* and *K_d_* can be derived by fitting *F* data *versus* total ligand concentration *L*_0_, using Equation (A.1). This equation has been used to fit the data of Figures 4 (red line) and 6A.

In the presence of denaturant, the fitting equation is the same, but all the fitting parameters are now labeled by a prime:

(A.2) $$F^{\prime }=F_{0}^{\prime }-\left(F_{0}^{\prime }-F_{\infty }^{\prime }\right)\frac{(K_{d}^{\prime }+P_{0}^{\prime }+L_{0})-\sqrt{{\left(K_{d}^{\prime }+P_{0}^{\prime }+L_{0}\right)}^{2}-4P_{0}^{\prime }L_{0}}}{2P_{0}^{\prime }}$$

### Ligand Fluorescence

Since in this case *F*_0_ ≈ 0, the fitting Equation (A.1) can be simplified as follows:

(A.3) $$F=F_{\infty }\frac{(K_{d}+P_{0}+L_{0})-\sqrt{{\left(K_{d}+P_{0}+L_{0}\right)}^{2}-4P_{0}L_{0}}}{2P_{0}}$$

This equation has been used to fit FRET data shown in Figure 4 (black line) as well as to determine *K_d_* from the AMA fluorescence excited at 350 nm, where no FRET is involved.

In the presence of denaturant, the fitting equation is the same as Equation (A.2), but since both *F*_0_ and *F*_0_’ are both negligible, it can be written as:

(A.4) $$\frac{F^{\prime }}{F_{\infty }}=\frac{(K_{d}^{\prime }+P_{0}^{\prime }+L_{0})-\sqrt{{\left(K_{d}^{\prime }+P_{0}^{\prime }L_{0}\right)}^{2}-4P_{0}^{\prime }L_{0}}}{2P_{0}}$$

These two equations have been used to fit the data shown in Figure 6B and to derive the corresponding *K_d_* values.
